# Outcome after liver resection for primary and recurrent intrahepatic cholangiocarcinoma

**DOI:** 10.1002/bjs5.50217

**Published:** 2019-09-10

**Authors:** A. Nickkholgh, O. Ghamarnejad, E. Khajeh, P. Tinoush, T. Bruckner, Y. Kulu, M. Mieth, B. Goeppert, S. Roessler, K. H. Weiss, K. Hoffmann, M. W. Büchler, A. Mehrabi

**Affiliations:** ^1^ Department of General, Visceral and Transplant Surgery Ruprecht‐Karls University Heidelberg Germany; ^2^ Institute of Medical Biometry and Informatics Ruprecht‐Karls University Heidelberg Germany; ^3^ Institute of Pathology Ruprecht‐Karls University Heidelberg Germany; ^4^ Department of Internal Medicine Ruprecht‐Karls University Heidelberg Germany; ^5^ Liver Cancer Centre Heidelberg Ruprecht‐Karls University Heidelberg Germany

## Abstract

**Background:**

Liver resection is the only curative therapeutic option for intrahepatic cholangiocarcinoma (ICC), but the approach to recurrent ICC is controversial. This study analysed the outcome of liver resection in patients with recurrent ICC.

**Methods:**

Demographic, radiological, clinical, operative, surgical pathological and follow‐up data for all patients with a final surgical pathological diagnosis of ICC treated in a tertiary referral centre between 2001 and 2015 were collected retrospectively and analysed.

**Results:**

A total of 190 patients had liver resection for primary ICC. The 1‐, 3‐ and 5‐year overall survival (OS) rates were 74·8, 56·6 and 37·9 per cent respectively. Independent determinants of OS were age 65 years or above (hazard ratio (HR) 2·18, 95 per cent c.i. 1·18 to 4·0; *P* = 0·012), median tumour diameter 5 cm or greater (HR 2·87, 1·37 to 6·00; *P* = 0·005), preoperative biliary drainage (HR 2·65, 1·13 to 6·20; *P* = 0·025) and local R1–2 status (HR 1·90, 1·02 to 3·53; *P* = 0·043). Recurrence was documented in 87 patients (45·8 per cent). The mean(s.d.) survival time after recurrence was 16(17) months. Independent determinants of recurrence were median tumour diameter 5 cm or more (HR 1·71, 1·09 to 2·68; *P* = 0·020), high‐grade (G3–4) tumour (HR 1·63, 1·04 to 2·55; *P* = 0·034) and local R1 status (HR 1·70, 1·09 to 2·65; *P* = 0·020). Repeat resection with curative intent was performed in 25 patients for recurrent ICC, achieving a mean survival of 25 (95 per cent c.i. 16 to 34) months after the diagnosis of recurrence. Patients deemed to have unresectable disease after recurrence received chemotherapy or chemoradiotherapy alone, and had significantly poorer survival.

**Conclusion:**

Patients with recurrent ICC may benefit from repeat surgical resection.

## Graphical abstract

The outcomes of liver resection in patients with intrahepatic cholangiocarcinoma in a tertiary referral centre were evaluated. Patients with recurrent intrahepatic cholangiocarcinoma may benefit from repeat surgery with curative intent.

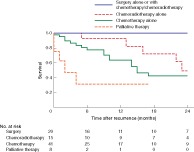

Surgery for recurrent iCCA may be beneficial.

## Abstract (es)

**Antecedentes:**

La resección hepática es la única opción terapéutica curativa para el colangiocarcinoma intrahepático (*intrahepatic colangiocarcinoma*, iCCA), pero el enfoque terapéutico de la recidiva del iCCA es controvertido. En este estudio se analizaron los resultados de la resección hepática en pacientes con recidiva de un iCCA.

**Métodos:**

Se recopilaron de forma retrospectiva y se analizaron los datos demográficos, radiológicos, clínicos, quirúrgicos, de anatomía patológica y de seguimiento de todos los pacientes con diagnóstico anatomopatológico definitivo de iCCA en un centro de referencia terciario entre 2001 y 2015.

**Resultados:**

En total, 190 pacientes se sometieron a resección hepática por iCCA primario. La supervivencia global (*overall survival*, OS) a 1, 3 y 5 años fue del 75%, 57% y 38%, respectivamente. La edad de ≥ 65 años (cociente de riesgos instantáneos, *hazard ratio*, HR 2,2, i.c. del 95% 1,2‐4,0, *P* = 0,012), la mediana del diámetro del tumor ≥ 5 cm (HR 2,9, i.c. del 95% 1,4‐6,0, *P* = 0,005), el drenaje biliar preoperatorio (HR 2,6, i.c. del 95% 1,3‐6,2, *P* = 0.025) y el estado local R1/2 (HR 1,9, i.c. del 95% 1,0‐3,5, *P* = 0,043) fueron factores pronósticos independientes de la OS. La recidiva se documentó en 87 (45,8%) pacientes. El tiempo medio de supervivencia después de la recidiva fue de 16 ± 2 meses. Los factores pronósticos independientes de recidiva fueron la mediana del diámetro del tumor ≥ 5 cm (HR 1,7, i.c. del 95% 1,1‐2,7, *P* = 0,020), el tumor de alto grado (G3‐G4) (HR 1,6, i.c. del 95% 1,0‐2,5, *P* = 0,034) y el estado local R1 (HR 1,7, i.c. del 95% 1,1‐2,6, *P* = 0,020). La resección repetida con intención curativa se realizó en 25 pacientes con iCCA recidivado, con una supervivencia media de 25 meses (i.c. del 95% 16‐34 meses) tras el diagnóstico de recidiva. Los pacientes que se consideraron no resecables después de la recidiva se sometieron a quimioterapia o quimiorradioterapia y presentaron una supervivencia significativamente peor.

**Conclusión:**

Los pacientes con recidiva de un iCCA pueden beneficiarse de la resección quirúrgica repetida.

## Introduction

Intrahepatic cholangiocarcinoma (ICC) originates from the secondary or smaller branches of the intrahepatic bile ducts. ICC presents predominantly with mass lesions of the liver, and accounts for 5–10 per cent of all cholangiocarcinomas^1^. It is a rare malignancy, with the exception of certain regions (in south‐east Asia) with well known predisposing epidemiological factors^2^. However, the recent increase in its incidence and mortality rate in several other geographical regions of the world has warranted a revisiting of the pathogenesis and therapeutic modalities^3^, ^4^.

Liver resection is the only curative treatment for patients with ICC. However, the majority of patients present with advanced unresectable disease, which precludes a potentially curative approach^5^. Moreover, the recurrence rate is high, reported to be 60 per cent or above^6^; management of recurrent ICC is challenging and may include resection or ablation^7^. The survival benefit of adjuvant therapies is unclear, and is currently under clinical investigation.

In the present cohort study of patients undergoing liver resection for ICC, the determinants of outcome were further characterized, with particular focus on revisiting the biological and surgical determinants of recurrence. The role of available modalities for managing recurrent disease was also evaluated.

## Methods

From a prospectively processed database of all patients undergoing liver resection at the Department of General, Abdominal and Transplant Surgery at the University Hospital of Heidelberg, all consecutive entries for patients who were referred and underwent liver resection for a new liver mass lesion between December 2001 and December 2015 were reviewed. Demographic, radiological, clinical, operative, surgical pathological and follow‐up data for all patients with a final surgical pathological diagnosis of ICC were collected. The ethics committee of the Faculty of Medicine at the Ruprecht‐Karls University of Heidelberg approved this retrospective study (S‐754/2018).

### Preoperative investigation

Evaluation of the patients included physical examination, blood tests for complete blood count and differentiation, biochemistry, liver function tests, coagulation studies, hepatitis serology, and the tumour markers alpha‐fetoprotein, carcinoembryonic antigen and carbohydrate antigen (CA) 19‐9. Contrast‐enhanced triple‐phase helical CT or MRI, or both (when necessary), were used in all patients to locate the lesion, the level of biliary obstruction, and the presence of lymphadenopathy. Magnetic resonance cholangiopancreatography, endoscopic retrograde cholangiopancreatography, percutaneous transhepatic cholangiopancreatography and, more recently, [^18^F]fluoro‐2‐deoxy‐d‐glucose‐PET, especially for detection of recurrence, were employed if necessary.

### Perioperative care and surgical technique

The institutional standards of hepatobiliary surgery have been published elsewhere^8^. Briefly, after laparotomy through a reversed L‐shaped incision, all patients underwent a thorough abdominal exploration to rule out metastatic disease, intraoperative ultrasonography of the liver and assessment of lymph nodes (LNs) of the hepatoduodenal ligament, retropancreatic and coeliac regions. Portal triad clamping (Pringle manoeuvre) was used when necessary. The extent of resection was defined for each patient according to the number, size and location of the lesion. The Brisbane 2000 nomenclature^9^ for liver anatomy and surgery was used to describe the liver resections. Transection of the liver parenchyma was performed under low central venous pressure (2–5 mmHg). The standard procedure for the parenchymal dissection was stapler hepatectomy^10^. *En bloc* resection of segment 1 (caudate lobectomy) and the resection (and reconstruction) of major vessels was done when necessary to achieve tumour clearance. Lymphadenectomy was performed in patients with lymphadenopathy. In patients without lymphadenopathy, LN sampling with frozen section was performed. Locoregional lymphadenectomy from the hepatoduodenal ligament, coeliac or posterior pancreatoduodenal regions was performed only in patients with positive findings on frozen‐section examination. Excision of the extrahepatic bile duct and reconstruction of bilioenteric continuity through a Roux‐en‐Y hepaticojejunostomy was performed if the biliary confluence was included in the resection. Argon‐beam coagulation and topical sealants were used liberally to stop bleeding at the resection surface. Abdominal drains were placed routinely. Transfusion of blood products was performed according to an established algorithm^11^. Postoperative biliary complications were graded according to the system of the International Study Group of Liver Surgery^12^.

### Pathological diagnosis

The diagnosis was confirmed in all patients by reviewing the clinical and histopathological data. Tumours were typed, graded and staged according to the eighth edition of the AJCC/UICC classification of malignant tumours. Tumours that could not be classified confidently according to the TNM classification were excluded. Patients with histological subtypes other than adenocarcinoma were excluded. Patients with unusual histological adenocarcinoma subtypes, such as papillary or mucinous adenocarcinoma and combined hepatocellular carcinoma/cholangiocarcinoma, were also excluded.

Additional immunohistological staining was performed in patients with untypical tumour histomorphology or in those with other known malignancies. Only patients with either a typical histomorphology or a typical immunohistochemistry for ICC (CK7, CK20 and CDX2) were included in the study. Adenocarcinomas of other origin in the medical record were accepted as primary to the liver only if they expressed CK7 with no expression of CK20, CDX2, or other markers of the known primary tumour..

### Statistical analysis

All variables that were potentially related to outcome were categorized into three main groups of preoperative, surgical pathological, and postoperative determinants of survival. IBM SPSS® Statistics version 25.0 for Windows® (IBM, Armonk, New York, USA) was used for statistical analysis. Data are presented as mean(s.d.) unless indicated otherwise. Overall survival (OS) was determined from the date of surgery to last follow‐up or death, whichever occurred first. Disease‐free survival (DFS) was calculated from the date of surgery to the date of recurrence, or to the date of death or last follow‐up. Receiver operating characteristic (ROC) curve analysis was used to determine cut‐off values for age, preoperative CA19‐9 level, preoperative total bilirubin level, and median tumour diameter for prediction of poor survival using Youden's *J* statistic. Survival rates were analysed with the Kaplan–Meier method, and differences were compared using the log rank test. Patients with a local R2 status after the primary resection were omitted from the DFS analysis. The influence of prognostic factors on outcome was assessed by means of Cox proportional hazard regression analysis, giving hazard ratios (HRs) and 95 per cent confidence intervals. Variables with *P* < 0·100 in the univariable analysis were included in the multivariable analysis. An effect was considered statistically significant at *P* < 0·050.

## Results

A total of 190 patients underwent liver resection for ICC. Demographic, preoperative, surgical and pathological data of patients undergoing liver resection are shown in *Table* 1. The only relevant predisposing factor identified in the past medical history of the patients was primary sclerosing cholangitis in nine patients (4·7 per cent). Nineteen patients (10·2 per cent) had undergone neoadjuvant chemotherapy with gemcitabine or 5‐fluorouracil and/or transarterial chemoembolization. The most frequent surgical approach entailed the resection of three or fewer segments, in 49 patients (25·8 per cent). Locoregional lymphadenectomy was performed in 91 of 189 patients (48·1 per cent) (median 2 (range 1–45) LNs).

**Table 1:** Demographic, preoperative, surgical and pathological data of patients with intrahepatic cholangiocarcinoma

	No. of patients* (*n* = 190)
**Age at diagnosis (years)** *	63 (24–86)
**Sex ratio (F** : **M)**	83 : 107
**Primary sclerosing cholangitis**	9 (4·7)
**Presenting signs/symptoms**	
Jaundice	41 of 187 (21·9)
Pain	69 of 163 (42·3)
Weight loss ≥ 5 kg	37 (19·5)
**Carbohydrate antigen 19‐9 level (units/ml)** *	32 (1–41 567)
**Bilirubin level (mg/dl)** *	0·8 (0·2–22·7)
**Preoperative biliary drainage**	17 (9·0)
**Neoadjuvant chemotherapy/TACE**	19 of 187 (10·2)
**PVE**	4 (2·1)
**Surgical procedure**	
Extended right hepatectomy	34 (17·9)
Extended left hepatectomy	21 (11·1)
Right hemihepatectomy	41 (21·6)
Left hemihepatectomy	45 (23·7)
Resection of ≤ 3 segments†	49 (25·8)
**Segment 1 resection**	70 of 188 (37·2)
**Bilioenteric anastomosis**	58 (30·5)
**Lymphadenectomy**	91 of 189 (48·1)
**(Partial) resection of major vessels**	55 (28·9)
**Tumour diameter (cm)** *	5·8 (0·2–21·0)
**Surgical margins**	*n* = 181
R0	117 (64·6)
R1	59 (32·6)
R2	5 (2·8)
**Tumour grade**	*n* = 176
G1	19 (10·8)
G2	101 (57·4)
G3	53 (30·1)
G4	3 (1·7)
**T category**	*n* = 182
T1	53 (29·1)
T2	68 (37·4)
T3	45 (24·7)
T4	16 (8·8)
**N category**	
Nx	63 (33·2)
N0	75 (39·5)
N1	52 (27·4)

With percentages in parentheses unless indicated otherwise;

* values are median (range).

† Includes three patients who had mesohepatectomy for liver segments 4, 5 and 8; excluding left hemihepatectomies. TACE, transarterial chemoembolization; PVE, portal vein embolization.

A clear resection margin (R0) was achieved in 117 (64·6 per cent) of 181 patients. Although not significantly different, patients with a positive resection margin (R1–2) had a larger tumour size than those with a clear margin (7 *versus* 6·1 cm respectively; *P* = 0·078). In addition, the rate of T3–4 status (46 *versus* 26·5 per cent; *P* = 0·012) and extended hepatectomy (42 *versus* 22·2 per cent; *P* = 0·006) was significantly higher in patients with an R1–2 resection margin than in those with a clear margin. Thirty of 188 patients (16·0 per cent) underwent relaparotomy for postoperative complications. The most common indication for relaparotomy was grade C bile leakage, in 15 patients (7·9 per cent). Median hospital stay was 14 (range 2–92) days. Thirteen patients (6·8 per cent) died within 30 days of surgery.

Adjuvant chemotherapy was performed in 57 of 187 patients (30·5 per cent). The protocol included gemcitabine in 27 patients, gemcitabine plus platinum in 18, FOLFOX‐3 (leucovorin, 5‐fluorouracil and oxaliplatin) in three and other protocols in nine patients. Twenty‐five of 187 patients (13·4 per cent) underwent adjuvant external‐beam radiotherapy (median dose 45 Gy). Median follow‐up was 19 (range 1–170) months.

### Survival analysis

The 1‐, 3‐ and 5‐year OS rates were 74·8, 56·6 and 37·9 per cent respectively. In the multivariable analysis for OS, age 65 years or over (HR 2·18, 95 per cent c.i. 1·18 to 4·03; *P* = 0·012), median tumour diameter of 5 cm or more (HR 2·87, 1·37 to 6·00; *P* = 0·005), preoperative biliary drainage (HR 2·65, 1·13 to 6·20; *P* = 0·025) and local R1–2 status (HR 1·90, 1·02 to 3·53; *P* = 0·043) were independent determinants of poor OS (*Table* *S1*, supporting information).

**Figure 1:**
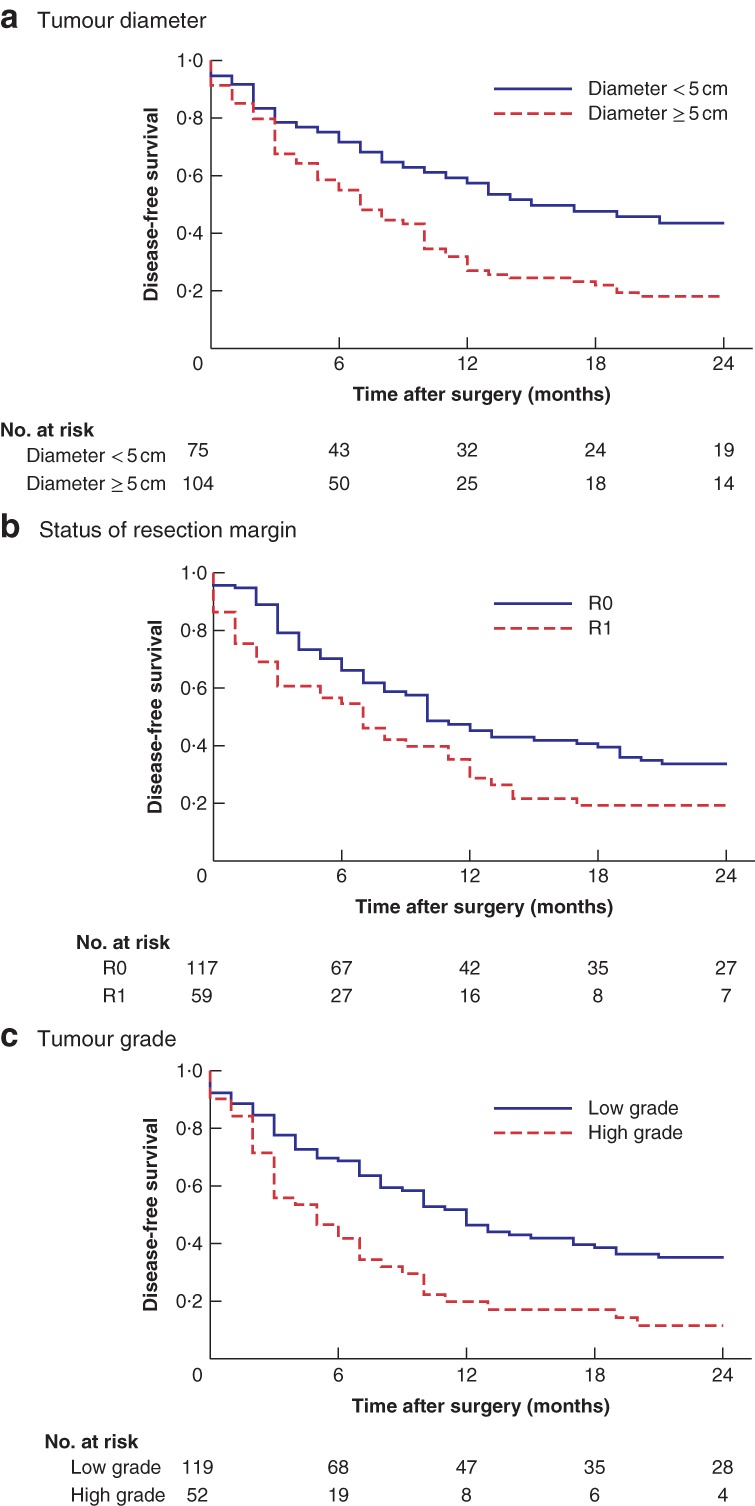
Kaplan–Meier plots analysis of 2‐year disease‐free survival Patients with: **a** tumour diameter of 5 cm or more *versus* those with tumour diameter of less than 5 cm; **b** R0 *versus* R1 resection margin; and **c** patients with low‐grade (G1–2) *versus* high‐grade (G3–4) tumour. The five patients with a local R2 status after primary resection were omitted from DFS analysis; data for tumour size, R status and tumour grade were not available for six, nine and 14 patients respectively. **a** *P* = 0·001, **b** *P* = 0·010, **c** *P* < 0·001 (log rank test).

In the multivariable analysis of DFS, independent predictive factors of poor DFS were median tumour diameter of 5 cm or greater (HR 1·71, 95 per cent c.i. 1·09 to 2·68; *P* = 0·020) and high‐grade (G3–4) tumour (HR 1·63, 1·04 to 2·55; *P* = 0·034) (*Table* *S2*, supporting information). Histological evidence of a positive tumour margin (local status R1) was the only surgical variable independently to predict significantly decreased DFS (HR 1·70, 1·09 to 2·65; *P* = 0·020). *Fig*. 1 depicts the Kaplan–Meier plots according to independent predictors of DFS.

### Recurrent intrahepatic cholangiocarcinoma

Recurrence was diagnosed in 87 of the 190 patients (45·8 per cent) during a median follow‐up of 10 (range 1–85) months. Fifty‐four patients had only an intrahepatic recurrence. Eighteen were diagnosed with only extrahepatic metastases on lymph nodes (6 patients), bone (3), mesenteric vessels (3), lung (2), peritoneum (2) or pancreas (2). Fifteen patients had both intrahepatic recurrence and extrahepatic metastases on lungs (7), lymph nodes (7) and peritoneum (1). The mean(s.d.) time to recurrence was 10(11) months, with a mean survival time after the documentation of recurrence of 16(17) months. Intervention‐associated survival after recurrence is shown in *Table* 2.

**Table 2:** Intervention‐associated survival after recurrence

Type of intervention after recurrence	No. of patients (*n* = 87)	Time to recurrence (months)*	Survival (months)*
**Exploration**	31 (36)	11(9) (7, 15)	22(21) (14, 29)
Resection†	25	11(8) (8, 15)	25(22) (16, 34)
Liver resection	20	11(9) (7, 15)	24(24) (13, 36)
Lymph node dissection	5	10(5) (3, 17)	28(10) (15, 40)
**Chemotherapy**	41‡ of 64 (64)	9(11) (6, 12)	13(14) (9, 18)
Gemcitabine monotherapy	15 of 41 (37)	10(15) (1, 18)	15(15) (6, 23)
Gemcitabine + platinum	19 of 41 (46)	9(8) (6, 13)	10(13) (4, 17)
FOLFOX‐3	3 of 41 (7)	7(6) (1, 22)	23(10) (1, 47)
Other	4 of 41 (10)	6(3) (1, 10)	14(15) (2, 38)
**Chemoradiotherapy**	15§ of 64 (23)	9(15) (1, 18)	16(11) (10, 22)
**RFA**	7§ (8)	7(5) (2, 13)	21(11) (10, 32)
**Palliative therapy**	8 of 87 (9)	18(14) (6, 30)	2(2) (0, 4)

Values in parentheses are percentages unless indicated otherwise;

* values are mean(s.d.) (95 per cent c.i.).

† Twenty‐one patients received additional chemotherapy/chemoradiotherapy after resection for recurrent intrahepatic cholangiocarcinoma.

‡ Excludes patients who had resection or chemoradiotherapy;

§ excludes patients who had resection. FOLFOX, leucovorin, 5‐fluorouracil and oxaliplatin; RFA, radiofrequency ablation.

#### *Management of recurrent disease*

The management of recurrence included (one or a combination of) exploration, chemotherapy, radiotherapy and radiofrequency ablation (RFA) (*Table* 2). There were no differences between different chemotherapeutic protocols used in the setting of recurrence (*P* = 0·738). Thirty‐one patients had surgery with curative intent for recurrence. Liver resection was performed in 20 patients (11 extra‐anatomical and 9 anatomical resections) and LN dissection in five patients. Six additional patients were deemed to have inoperable disease during surgery and underwent biopsy. Forty‐one patients received chemotherapy alone for recurrence. The chemotherapeutic protocol was gemcitabine monotherapy in 15 patients, gemcitabine plus platinum chemotherapy in 19 and FOLFOX‐3 regimen in three. Combined gemcitabine‐based chemoradiotherapy was also performed in 15 patients. RFA was performed in combination with either surgery (9 patients), chemotherapy/chemoradiotherapy (4) or alone (3).

#### *Repeat liver resection for recurrent intrahepatic cholangiocarcinoma*

**Figure 2:**
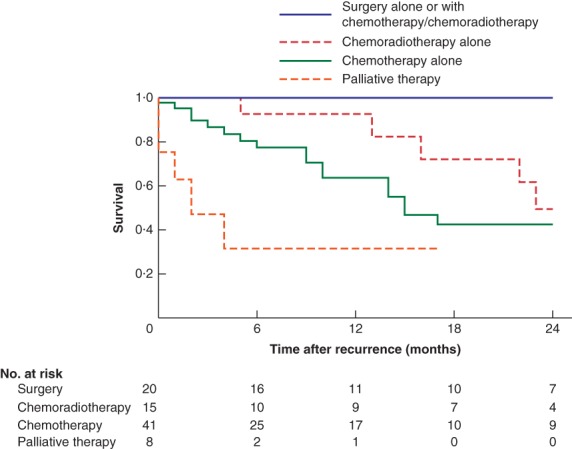
Kaplan–Meier analysis of 2‐year survival after recurrence of intrahepatic cholangiocarcinoma Thirty‐one patients had a repeat operation with curative intent for recurrence; liver resection was performed in 20 patients and lymph node dissection in five. Additional chemotherapy/chemoradiotherapy was performed in 21 patients after resection. Chemotherapy alone was received by 41 patients, and chemoradiotherapy alone by 15. Eight patients had standard palliative care only. Radiofrequency ablation (RFA) was performed in combination with either surgery (9 patients), chemotherapy/chemoradiotherapy (4) or alone (3); patients who had only RFA were not included in the survival analysis. Patients undergoing resection had better survival (*P* < 0·001, log rank test).

OS varied significantly between different groups of patients who underwent resection (with or without chemotherapy or chemoradiotherapy), chemoradiotherapy alone, or chemotherapy alone for recurrent disease (*P* < 0·001) (*Fig*. 2). Anatomical monosegmentectomy was performed in seven patients, non‐anatomical monosegmentectomy in seven, anatomical bisegmentectomy in three, non‐anatomical bisegmentectomy in two, and tumour excision in one patient after recurrence of ICC. Detailed perioperative data for the 20 patients who had repeat liver resection for recurrent ICC are shown in *Table* 3.

**Table 3:** Details of 20 patients who had repeat liver resection for recurrent intrahepatic cholangiocarcinoma

Patient no.	TNM grade	Resection margin	Type of primary liver resection	Adjuvant therapy	Time to recurrence (months)	Location of recurrence (segments)	Type of repeat liver resection	Additional therapy	Follow‐up after recurrence (months)
1	T3 N0 M0 G2	R0	Right EH	No	3	2 + 3	Non‐anatomical segments	Radiotherapy	33*
2	T3 Nx M0 G1	R0	Minor (< 3 segments)	Yes	3	2 + 3	Anatomical segments	Gemcitabine	11
3	T1 N0 M0 G1	R0	Left HH	No	11	5 + 7	Non‐anatomical segments	Gemcitabine + cisplatin	41*
4	T3 N1 M0 G3	R1	Minor (< 3 segments)	Yes	5	4	Anatomical segment	Capecitabine	1
5	T2 Nx M0 G3	R0	Right HH	No	10	4	Anatomical segment	FOLFOX‐3	76
6	T2 N0 M0 G2	R1	Left EH	Yes	5	7	Anatomical segment	–	19
7	T1 N0 M0 G3	R0	Right EH	No	8	2	Non‐anatomical segment	Gemcitabine	53
8	T1 Nx M0 G1	R0	Minor (< 3 segments)	No	5	8	Anatomical segment	–	85
9	T1 Nx M0 G1	R0	Minor (< 3 segments)	No	15	2 + 3	Anatomical segments	Cetuximab + radiotherapy	21
10	T2 N0 M0 G2	R0	Left EH	No	8	7	Anatomical segment	Gemcitabine + cisplatin	16
11	T3 Nx M0 G3	R1	Right EH	No	11	2	Tumour excision	Gemcitabine + cisplatin	39*
12	T2 Nx M0 G3	R0	Minor (< 3 segments)	No	7	7 + 8	Anatomical segments	Gemcitabine + cisplatin	11
13	T1 Nx M0 G2	R0	Minor (< 3 segments)	No	6	4	Anatomical segment	Gemcitabine + cisplatin	3
14	T2 N0 M0 G1	R0	Right HH	No	17	4	Non‐anatomical segment	Gemcitabine + radiotherapy	9
15	T1 Nx M0 G1	R1	Right EH	No	40	2	Anatomical segment	Gemcitabine + cisplatin	23
16	T4 N1 M0 G3	R1	Left HH	No	6	5	Non‐anatomical segment	Gemcitabine + cisplatin	7
17	T2 Nx M0 G3	R0	Right HH	No	21	4	Non‐anatomical segment	Capecitabine	4
18	T1 Nx M0 G2	R1	Left EH	No	17	6	Non‐anatomical segment	–	26
19	T3 Nx M0 G2	R0	Right HH	Yes	19	4	Non‐anatomical segment	–	1
20	T1 Nx M0 G2	R0	Left HH	No	12	8	Non‐anatomical segment resection	Gemcitabine + cisplatin	8

* Died at end of follow‐up from disseminated intrahepatic cholangiocarcinoma. EH, extended hepatectomy; HH, hemihepatectomy; FOLFOX, leucovorin, 5‐fluorouracil and oxaliplatin.

## Discussion

In this study, several different preoperative, surgical and clinicopathological variables were associated with reduced OS (age 65 years or more, median tumour diameter of 5 cm or greater, preoperative biliary drainage and local R1–2 status) and DFS (median tumour diameter 5 cm or more, high‐grade (G3–4) tumour and local R1 status). In the present cohort, a R0 rate of almost 65 per cent, a recurrence rate of less than 46 per cent, and a 5‐year OS rate of almost 40 per cent were achieved. The high positive resection margin rate in the present study could be explained by the large number of patients with an advanced tumour stage, who were treated in the tertiary institution. In addition, the present study has shown that the cohort of patients having repeat surgery with curative intent, with or without additional therapy, for recurrent ICC has a significantly better outcome than those undergoing chemotherapy or chemoradiotherapy alone.

Despite advances in understanding the pathophysiology of ICC and the emergence of novel therapeutic options^13^, liver resection remains the only chance of cure in patients with ICC, with no significant improvement in survival in recent decades^14^, ^15^, ^16^. The management of recurrent ICC has drawn increasing attention recently, with some studies^17^, ^18^, ^19^, ^20^ with relatively larger numbers of patients helping to delineate better the role of repeat resection for recurrent ICC. These cohorts, however, lack sufficient numbers of patients to be able to define precisely the role of surgery, as well as that of additional therapy, in recurrent ICC. The present cohort of patients with recurrent ICC undergoing repeat surgery with curative intent was almost as large as that from two recent multi‐institutional studies^18^, ^20^. Furthermore, the results of the present study are similar to the findings of Si and colleagues^18^, who reported on the largest cohort of patients (72) undergoing repeat liver resection, in which the median OS time was 45·1 months with a 1‐year survival rate of 97 per cent.

A study^21^ of patients with ICC from Memorial Sloan‐Kettering Cancer Center in New York reported the presence of multiple hepatic tumours as the only independent predictor of both disease‐free and disease‐specific survival. These authors showed that tumour diameter of 5 cm or less was significantly associated with longer recurrence‐free survival, but this association was lost in the multivariable analysis. It was shown that a median tumour diameter of 5 cm or more could independently predict both poor OS and DFS, in line with the findings from more recent cohorts^19^, ^20^.

Using SEER (Surveillance, Epidemiology and End Results) data, it has been shown^22^ that node positivity is associated with worse survival in patients with cholangiocarcinoma. Ito and co‐workers^23^ showed a disease‐specific survival benefit for patients with N0 disease with hilar cholangiocarcinoma in whom a minimum of seven lymph nodes had been removed. Although locoregional lymphadenectomy has been recommended for both extrahepatic cholangiocarcinoma^24^ and gallbladder cancer^25^, the clinical benefit of lymphadenectomy in ICC is unknown, and it is not a widely performed component of surgical resection for the condition, especially in Western medical centres^26^. In this study, a statistically prognostic significance for the locoregional lymphadenectomy in ICC could not be proved.

The most important limitation of this study, like that of other similar studies, is the low sample size of the patients with recurrent ICC for whom a definitive therapeutic plan can be devised. Even in more recent, relatively larger, cohorts of patients with recurrent ICC, the number undergoing definitive treatment was still too low to allow any significant conclusion regarding the role of surgery and additional therapy in this setting. A further limitation of this study is the retrospective design, which may result in varied durations of follow‐up, as well as loss to follow‐up. Well designed, larger, international multi‐institutional studies are needed for more precise conclusions to be drawn.

OS and DFS rates following liver resection for ICC remain poor. The recurrence rate is high, especially in the setting of bigger tumours (5 cm or larger), higher grade (G3–4) and local tumour‐positive resection margin (R1 status) despite adjuvant therapy. The approach to recurrent disease is challenging, although patients seem to have survival benefit from repeat surgery. More effective additional strategies are clearly needed, and should be investigated in multicentre clinical trials.

## Disclosure

The authors declare no conflict of interest.

## Supporting information


**Table S1.** Univariable and multivariable analysis of predictors of overall survival
**Table S2.** Univariable and multivariable analysis of predictors of disease‐free survival

## References

[1] Hirohashi K , Uenishi T , Kubo S , Yamamoto T , Tanaka H , Shuto T *et al* Macroscopic types of intrahepatic cholangiocarcinoma: clinicopathologic features and surgical outcomes. Hepatogastroenterology 2002; 49: 326–329. 11995443

[2] Shaib Y , El‐Serag HB . The epidemiology of cholangiocarcinoma. Semin Liver Dis 2004; 24: 115–125. 15192785 10.1055/s-2004-828889

[3] Patel T. Increasing incidence and mortality of primary intrahepatic cholangiocarcinoma in the United States. Hepatology 2001; 33: 1353–1357. 11391522 10.1053/jhep.2001.25087

[4] Taylor‐Robinson SD , Toledano MB , Arora S , Keegan TJ , Hargreaves S , Beck A *et al* Increase in mortality rates from intrahepatic cholangiocarcinoma in England and Wales 1968–1998. Gut 2001; 48: 816–820. 11358902 10.1136/gut.48.6.816 PMC1728314

[5] Hammad AY , Berger NG , Eastwood D , Tsai S , Turaga KK , Christian KK *et al* Is radiotherapy warranted following intrahepatic cholangiocarcinoma resection? The impact of surgical margins and lymph node status on survival. Ann Surg Oncol 2016; 23(Suppl 5): 912–920. 27654107 10.1245/s10434-016-5560-1

[6] Weber SM , Jarnagin WR , Klimstra D , DeMatteo RP , Fong Y , Blumgart LH . Intrahepatic cholangiocarcinoma: resectability, recurrence pattern, and outcomes. J Am Coll Surg 2001; 193: 384–391. 11584966 10.1016/s1072-7515(01)01016-x

[7] Bridgewater J , Galle PR , Khan SA , Llovet JM , Park JW , Patel T *et al* Guidelines for the diagnosis and management of intrahepatic cholangiocarcinoma. J Hepatol 2014; 60: 1268–1289. 24681130 10.1016/j.jhep.2014.01.021

[8] Reissfelder C , Rahbari NN , Koch M , Kofler B , Sutedja N , Elbers H *et al* Postoperative course and clinical significance of biochemical blood tests following hepatic resection. Br J Surg 2011; 98: 836–844. 21456090 10.1002/bjs.7459

[9] Strasberg SM . Nomenclature of hepatic anatomy and resections: a review of the Brisbane 2000 system. J Hepatobiliary Pancreat Surg 2005; 12: 351–355. 16258801 10.1007/s00534-005-0999-7

[10] Schemmer P , Friess H , Hinz U , Mehrabi A , Kraus TW , Z'Graggen K *et al* Stapler hepatectomy is a safe dissection technique: analysis of 300 patients. World J Surg 2006; 30: 419–430. 16467982 10.1007/s00268-005-0192-9

[11] Rahbari NN , Elbers H , Koch M , Bruckner T , Vogler P , Striebel F *et al* Clamp‐crushing *versus* stapler hepatectomy for transection of the parenchyma in elective hepatic resection (CRUNSH) – a randomized controlled trial (NCT01049607). BMC Surg 2011; 11: 22. 21888669 10.1186/1471-2482-11-22 PMC3177759

[12] Koch M , Garden OJ , Padbury R , Rahbari NN , Adam R , Capussotti L *et al* Bile leakage after hepatobiliary and pancreatic surgery: a definition and grading of severity by the International Study Group of Liver Surgery. Surgery 2011; 149: 680–688. 21316725 10.1016/j.surg.2010.12.002

[13] Shiao MS , Chiablaem K , Charoensawan V , Ngamphaiboon N , Jinawath N . Emergence of intrahepatic cholangiocarcinoma: how high‐throughput technologies expedite the solutions for a rare cancer type. Front Genet 2018; 9: 309. 30158952 10.3389/fgene.2018.00309 PMC6104394

[14] Bagante F , Spolverato G , Weiss M , Alexandrescu S , Marques HP , Aldrighetti L *et al* Impact of morphological status on long‐term outcome among patients undergoing liver surgery for intrahepatic cholangiocarcinoma. Ann Surg Oncol 2017; 24: 2491–2501. 28466403 10.1245/s10434-017-5870-y

[15] de Jong MC , Nathan H , Sotiropoulos GC , Paul A , Alexandrescu S , Marques H *et al* Intrahepatic cholangiocarcinoma: an international multi‐institutional analysis of prognostic factors and lymph node assessment. J Clin Oncol 2011; 29: 3140–3145. 21730269 10.1200/JCO.2011.35.6519

[16] Okabayashi T , Yamamoto J , Kosuge T , Shimada K , Yamasaki S , Takayama T *et al* A new staging system for mass‐forming intrahepatic cholangiocarcinoma: analysis of preoperative and postoperative variables. Cancer 2001; 92: 2374–2383. 11745293 10.1002/1097-0142(20011101)92:9<2374::aid-cncr1585>3.0.co;2-l

[17] Park HM , Yun SP , Lee EC , Lee SD , Han SS , Kim SH *et al* Outcomes for patients with recurrent intrahepatic cholangiocarcinoma after surgery. Ann Surg Oncol 2016; 23: 4392–4400. 27581609 10.1245/s10434-016-5454-2

[18] Si A , Li J , Xing X , Lei Z , Xia Y , Yan Z *et al* Effectiveness of repeat hepatic resection for patients with recurrent intrahepatic cholangiocarcinoma: factors associated with long‐term outcomes. Surgery 2017; 161: 897–908. 27989605 10.1016/j.surg.2016.10.024

[19] Souche R , Addeo P , Oussoultzoglou E , Herrero A , Rosso E , Navarro F *et al* First and repeat liver resection for primary and recurrent intrahepatic cholangiocarcinoma. Am J Surg 2016; 212: 221–229. 26552996 10.1016/j.amjsurg.2015.07.016

[20] Yamashita YI , Shirabe K , Beppu T , Eguchi S , Nanashima A , Ohta M *et al* Surgical management of recurrent intrahepatic cholangiocarcinoma: predictors, adjuvant chemotherapy, and surgical therapy for recurrence: a multi‐institutional study by the Kyushu Study Group of Liver Surgery. Ann Gastroenterol Surg 2017; 1: 136–142. 29863136 10.1002/ags3.12018 PMC5881338

[21] Endo I , Gonen M , Yopp AC , Dalal KM , Zhou Q , Klimstra D *et al* Intrahepatic cholangiocarcinoma: rising frequency, improved survival, and determinants of outcome after resection. Ann Surg 2008; 248: 84–96. 18580211 10.1097/SLA.0b013e318176c4d3

[22] Nathan H , Aloia TA , Vauthey JN , Abdalla EK , Zhu AX , Schulick RD *et al* A proposed staging system for intrahepatic cholangiocarcinoma. Ann Surg Oncol 2009; 16: 14–22. 18987916 10.1245/s10434-008-0180-z

[23] Ito K , Ito H , Allen PJ , Gonen M , Klimstra D , D'Angelica MI *et al* Adequate lymph node assessment for extrahepatic bile duct adenocarcinoma. Ann Surg 2010; 251: 675–681. 20224368 10.1097/SLA.0b013e3181d3d2b2

[24] Mulholland MW , Yahanda A , Yeo CJ . Multidisciplinary management of perihilar bile duct cancer. J Am Coll Surg 2001; 193: 440–447. 11584972 10.1016/s1072-7515(01)01029-8

[25] Noshiro H , Chijiiwa K , Yamaguchi K , Shimizu S , Sugitani A , Tanaka M . Factors affecting surgical outcome for gallbladder carcinoma. Hepatogastroenterology 2003; 50: 939–944. 12845954

[26] Clark CJ , Wood‐Wentz CM , Reid‐Lombardo KM , Kendrick ML , Huebner M , Que FG . Lymphadenectomy in the staging and treatment of intrahepatic cholangiocarcinoma: a population‐based study using the National Cancer Institute SEER database. HPB (Oxford) 2011; 13: 612–620. 21843261 10.1111/j.1477-2574.2011.00340.x PMC3183445

