# Supplementary material for: Outcome after liver resection for primary and recurrent intrahepatic cholangiocarcinoma
Source: BJS Open. 2019 Sep 10;3(6):793–801. doi: 10.1002/bjs5.50217 (PMC6887914; doi:10.1002/bjs5.50217)
Supplement: Supplementary file 1 — Table S1. Univariable and multivariable analysis of predictors of overall survival Table S2. Univariable and multivariable analysis of predictors of disease‐free survival [file BJS5-3-793-s001.docx]

**BJS5_50217**

**Outcome after liver resection for primary and recurrent intrahepatic cholangiocarcinoma**

**A. Nickkholgh, O. Ghamarnejad, E. Khajeh, P. Tinoush, T. Bruckner, Y. Kulu, M. Mieth, B. Goeppert, S. Roessler, K. H. Weiss, K. Hoffmann, M. W. Büchler and A. Mehrabi**

| **Table S1** Univariable and multivariable analysis of predictors of overall survival | | | | | |
| --- | --- | --- | --- | --- | --- |
| Variables | n | mean survival ± SEM (mo), (95% CI) | *P*  univariate | Hazard Ratio  (95% CI) | *P*  Multivariate |
| Gender  Male  Female^*^ | 107  83 | 18 ± 3 (13-23)  27 ± 3 (21-33) | 0.312 |  |  |
| Age at diagnosis  ≥65 yr  ˂65 yr^*^ | 81  109 | 15 ± 3 (10-21)  27 ± 3 (22-33) | 0.011 | 2.183 (1.184-4.026) | **0.012** |
| Primary sclerosing cholangitis  Yes  No^*^ | 9  181 | 18 ± 6 (4-31)  22 ± 2 (18-27) | 0.354 |  |  |
| Jaundice  Present  Absent^*^ | 41  146 | 12 ± 3 (6-17)  25 ± 2 (21-30) | <0.001 | 1.195 (0.433-3.297) | 0.731 |
| Preoperative CA 19-9 level  ≥ 86 u/mL  ˂ 86 u/mL^*^ | 52  128 | 13 ± 2 (8-17)  26 ± 3 (20-31) | 0.001 | 1.453 (0.770-2.741) | 0.249 |
| Preoperative total bilirubin level  ≥ 1.2 mg/dL  ˂ 1.2 mg/dL^*^ | 64  123 | 15 ± 3 (10-21)  26 ± 3 (20-31) | 0.002 | 1.975 (0.919-4.243) | 0.081 |
| Preoperative biliary drainage  Present  Absent^*^ | 17  171 | 16 ± 5 (4-27)  23 ± 2 (19-27) | 0.005 | 2.650 (1.133-6.200) | **0.025** |
| Neoadjuvant chemotherapy/TACE  Yes  No^*^ | 19  168 | 15 ± 4 (8-23)  23 ± 2 (19-28) | 0.988 |  |  |
| Surgical procedure  Major hepatectomy (> 3 segments)  Minor hepatectomy (≤ 3 segments)^*^ | 141  49 | 20 ± 2 (16-24)  27 ± 5 (17-38) | 0.016 | 1.364 (0.587-3.169) | 0.470 |
| Bilioenteric anastomosis  Yes  No^*^ | 58  132 | 13 ± 2 (8-17)  26 ± 3 (21-32) | <0.001 | 1.075 (0.462-2.499) | 0.867 |
| Lymphadenectomy  Yes  No^*^ | 91  98 | 20 ± 2 (15-24)  25 ± 3 (18-31) | 0.856 |  |  |
| (Partial) resection of major vessels  Yes  No^*^ | 55  135 | 21 ± 3 (14-27)  23 ± 3 (18-28) | 0.241 |  |  |
| Median tumor diameter  ≥ 5 cm  ˂ 5 cm^*^ | 106  78 | 21 ± 3 (16-27)  24 ± 3 (18-30) | 0.022 | 2.870 (1.372-6.005) | **0.005** |
| Surgical margins  R0^*^  R1-2 | 117  64 | 28 ± 3 (22-34)  14 ± 2 (10-17) | <0.001 | 1.896 (1.020-3.526) | **0.043** |
| Tumor grading  Low-grade (G1-G2)^*^  High-grade (G3-G4) | 120  56 | 25 ± 3 (20-31)  17 ± 3 (11-23) | 0.053 | 1.123 (0.618-2.038) | 0.704 |
| T-Stage  T1^*^  T2  T3  T4 | 53  68  45  16 | 34 ± 5 (24-45)  20 ± 3 (15-26)  17 ± 3 (11-23)  17 ± 3 (11-23) | <0.001 | 1.252 (0.906-1.728) | 0.173 |
| N-Stage  Nx^*^  N0  N1 | 63  52  75 | 25 ± 4 (18-32)  27 ± 4 (20-34)  11 ± 2 (7-16) | 0.938 |  |  |
| Adjuvant chemotherapy  Yes  No^*^  Adjuvant radiotherapy  Yes  No^*^ | 57  130  25  161 | 15 ± 2 (11-19)  26 ± 3 (20-31)  17 ± 4 (10-25)  24 ± 2 (19-28) | 0.965  0.747 |  |  |
| SEM: standard error of mean; mo: month  ^*^  Reference parameters | | | | | |

| **Table S2** Univariable and multivariable analysis of predictors of disease-free survival | | | | | |
| --- | --- | --- | --- | --- | --- |
| Variables | n | mean survival ±  SEM (mo), (95% CI) | *P*  univariate | Hazard Ratio  (95% CI) | *P*  Multivariate |
| Gender^†^  Male  Female^*^ | 106  79 | 13 ± 2 (9-18)  18 ± 3 (12-24) | 0.248 |  |  |
| Age at diagnosis  ≥65 yr  ˂65 yr^*^ | 80  105 | 12 ± 3 (7-17)  18 ± 3 (12-23) | 0.175 |  |  |
| Primary sclerosing cholangitis  Yes  No^*^ | 9  176 | 13 ± 6 (1-27)  15 ± 2 (11-19) | 0.432 |  |  |
| Jaundice  Present  Absent^*^ | 40  142 | 8 ± 2 (4-13)  17 ± 2 (13-22) | 0.106 |  |  |
| Preoperative CA 19-9 level  ≥ 86 u/mL  ˂ 86 u/mL^*^ | 49  126 | 7 ± 2 (4-10)  18 ± 3 (13-23) | 0.006 | 1.454 (0.913-2.315) | 0.115 |
| Preoperative total bilirubin level  ≥ 1.2 mg/dL  ˂ 1.2 mg/dL^*^ | 63  120 | 10 ± 2 (5-15)  18 ± 3 (13-23) | 0.049 | 1.416 (0.894-2.244) | 0.138 |
| Preoperative biliary drainage  Present  Absent^*^ | 16  167 | 13 ± 5 (3-23)  16 ± 2 (12-20) | 0.362 |  |  |
| Neoadjuvant chemotherapy/TACE  Yes  No^*^ | 19  163 | 9 ± 3 (2-15)  16 ± 2 (12-20) | 0.275 |  |  |
| Surgical procedure  Major hepatectomy (> 3 segments)  Minor hepatectomy (≤ 3 segments) ^*^ | 138  47 | 14 ± 2 (10-17)  20 ± 5 (9-31) | 0.279 |  |  |
| Bilioenteric anastomosis  Yes  No^*^ | 56  129 | 10 ± 2 (6-14)  18 ± 3 (13-23) | 0.242 |  |  |
| Lymphadenectomy  Yes  No^*^ | 88  96 | 13 ± 2 (9-17)  17 ± 3 (11-23) | 0.545 |  |  |
| (Partial) resection of major vessels  Yes  No^*^ | 54  131 | 13 ± 3 (8-18)  16 ± 2 (11-21) | 0.741 |  |  |
| Median tumor diameter  ≥ 5 cm  ˂ 5 cm^*^ | 104  75 | 14 ± 3 (9-19)  18± 3 (12-24) | 0.006 | 1.706 (1.087-2.677) | **0.020** |
| Surgical margins  R0^*^  R1 | 117  64 | 20 ± 3 (14-25)  8 ± 1 (5-11) | 0.002 | 1.697 (1.087-2.650) | **0.020** |
| Tumor grading  Low-grade (G1-G2) ^*^  High-grade (G3-G4) | 119  52 | 19 ± 3 (14-24)  9 ± 2 (4-13) | 0.001 | 1.626 (1.038-2.545) | **0.034** |
| T-Stage  T1^*^  T2  T3  T4 | 53  65  43  16 | 25 ± 5 (15-35)  15 ± 3 (10-20)  9 ± 3 (4-15)  5 ± 1 (2-7) | <0.001 | 1.223 (0.978-1.530) | 0.078 |
| N-Stage  Nx^*^  N0  N1 | 63  52  70 | 16 ± 3 (10-23)  20 ± 4 (13-27)  6 ± 1 (3-8) | 0.194 |  |  |
| Adjuvant chemotherapy  Yes  No^*^  Adjuvant radiotherapy  Yes  No^*^ | 53  129  22  159 | 8 ± 1 (5-11)  18 ± 3 (13-24)  10 ± 3 (4-17)  16 ± 2 (12-20) | 0.215  0.250 |  |  |
| ^†^: patients with a local R2 status after the primary resection (n=5) were omitted from DFS analysis.  SEM: standard error of mean; mo: month  ^*^  Reference parameters | | | | | |
